# MicroRNAs as potential biomarkers for the diagnosis of Traumatic Brain Injury: A systematic review and meta-analysis

**DOI:** 10.7150/ijms.48214

**Published:** 2021-01-01

**Authors:** Qian Zhou, Jian Yin, Yadong Wang, Xinsuo Zhuang, Zhiyang He, Zuobing Chen, Xiaofeng Yang

**Affiliations:** 1 Department of Emergency and Trauma Center, The International Medical Center, The First Affiliated Hospital, College of Medicine, Zhejiang University, 79 Qingchun Road, Hangzhou 310003, Zhejiang Province, China.; 2 Department of Rehabilitation Medicine, The First Affiliated Hospital, Zhejiang University, 79 Qingchun Road, Hangzhou 310003, Zhejiang Province, China.

**Keywords:** miRNAs, TBI, diagnosis, biomarker, meta-analysis

## Abstract

**Background:** Traumatic brain injury (TBI) is a sudden trauma on the head, in which severe TBI (sTBI) is usually associated with death and long-term disability. MicroRNAs (miRNAs) are potential biomarkers of diverse diseases, including TBI. However, few systematic reviews and meta-analyses have been conducted to determine the clinical value of miRNAs expression in TBI patients.

**Methods:** We conducted this systematic review and meta-analysis study according to Preferred Reporting Items for Systematic Reviews and Meta-Analyses (PRISMA) guidelines. We searched PubMed, Embase, the Cochrane Library, Web of Science, from inception to August 26, 2020. We included articles written in English that have reported on the diagnostic value of miRNAs expression in TBI patients. We excluded studies that did not provided sufficient information to construct the 2×2 contingency table.

**Results:** Eight studies investigating the diagnostic value of miRNA in TBI were analyzed in this study. The overall sensitivity, specificity and area under the curve (AUC) of miRNAs in diagnosis of TBI were 89% [95% confidence interval (CI): 0.84-0.93], 92% (95% CI 0.82-0.97) and 95% (95% CI 0.93-0.97). We found that panels of multiple miRNAs could improve the diagnostic accuracy of TBI. Samples from blood and brain tissue have significantly enhanced diagnostic accuracy, when compared with saliva. The AUC of miRNAs in severe TBI was 0.97, with 91% sensitivity and 92% specificity.

**Conclusion:** This systematic review and meta-analysis demonstrated that miRNAs could be potential diagnostic markers in TBI patients. MiRNAs detected in blood and brain tissue display high accuracy for TBI diagnosis.

## Introduction

Traumatic brain injury (TBI) is a prevalent form of nervous system ailment that inflicts more than 50 million people each year worldwide 1. TBI, particularly severe TBI (sTBI), cause an enormous socio-economic and health care burden because of its associated mortalities and long-term disability among patients. Due to their clinical value in the diagnosis, prognosis and treatment of TBI patients, biomarkers have attracted considerable attention 2.

MicroRNAs (miRNAs) are a class of small non-coding RNAs with a length of 19-22 nucleotides. Many studies have found that miRNAs play a significant role in the maintenance and regulation of physiological function in TBI 3. Recently, the expression of miRNAs in TBI has been extensively examined. Many studies have revealed that some miRNAs have diagnostic and prognostic value in TBI. For example, Pietrao et al. showed that miR-425-5p is significantly downregulated in mild TBI (mTBI) and is a good diagnostic and prognostic indicator for TBI 4. Furthermore, Redell et al. detected substantial plasma quantities of miR-16, miR-92a, and miR-765 in sTBI patients, and found that the miRNAs have high diagnostic value in sTBI 5. Another study proved that miR-3195 and miR-328-5p may be used to distinguish mild and moderate TBI from sTBI 6. These studies have shown that miRNA can be used to diagnose TBI, including distinguishing sTBI from healthy controls and mild to moderate TBI. However, there are no meta-analyses of the clinical values of miRNAs in TBI patients. In this study, we conducted a meta-analysis to identify the potential diagnostic values of miRNAs in TBI patients. The purpose of this study was to explore the key miRNAs and provide useful information and direction for future study in the clinical value of miRNAs in TBI, such as prognosis of outcome.

## Materials and Methods

### Search strategy

Relevant studies published before August 26, 2020 was comprehensively searched through the English databases PubMed, Cochrane Library, Web of Science, and EMBASE. We used “TBI” and “miRNA” as the main key words and the following strategy to search PubMed: (((“Brain Injuries, Traumatic”[Mesh]) OR (((((((((((((((Brain Injury, Traumatic[Title/Abstract]) OR Traumatic Brain Injuries[Title/Abstract]) OR Trauma, Brain[Title/Abstract]) OR Brain Trauma[Title/Abstract]) OR Brain Traumas[Title/Abstract]) OR Traumas, Brain[Title/Abstract]) OR TBI (Traumatic Brain Injury)[Title/Abstract]) OR Encephalopathy, Traumatic[Title/Abstract]) OR Encephalopathies, Traumatic[Title/Abstract]) OR Traumatic Encephalopathies[Title/Abstract]) OR Injury, Brain, Traumatic[Title/Abstract]) OR Traumatic Encephalopathy[Title/Abstract]) OR TBIs (Traumatic Brain Injuries)[Title/Abstract]) OR TBI (Traumatic Brain Injuries)[Title/Abstract]) OR Traumatic Brain Injury[Title/Abstract]))) AND ((“MicroRNAs”[Mesh]) OR (((((((((((((((((MicroRNA[Title/Abstract]) OR miRNAs[Title/Abstract]) OR Micro RNA[Title/Abstract]) OR RNA, Micro[Title/Abstract]) OR miRNA[Title/Abstract]) OR Primary MicroRNA[Title/Abstract]) OR MicroRNA, Primary[Title/Abstract]) OR Primary miRNA[Title/Abstract]) OR miRNA, Primary[Title/Abstract]) OR pri-miRNA[Title/Abstract]) OR pri miRNA[Title/Abstract]) OR RNA, Small Temporal[Title/Abstract]) OR Temporal RNA, Small[Title/Abstract]) OR stRNA[Title/Abstract]) OR Small Temporal RNA[Title/Abstract]) OR pre-miRNA[Title/Abstract]) OR pre miRNA[Title/Abstract])).

### Eligibility criteria and Data extraction

The inclusion criteria are as follows: (1) articles provided diagnostic capacity of miRNA for TBI; (2) articles provided enough data such as true positives (TP), false positives (FP), false negatives (FN), true negatives (TN) or sensitivity and specificity to construct the 2×2 contingency table; (3) Publications written in English. The exclusion criteria are as follows: (1) Non-TBI or miRNAs researches; (2) Non-English Articles; (3) Animal or cell experiments; (4) meeting records, reviews and letters.

We extracted the following data according to our previous protocol: the first author's name; study population, sample sizes and regions; year of publication; and the false and true positives and negatives 7.

### Statistical analysis

Two reviewers (Zhou and Yin) extracted the number of TP, FP, FN and TN, which was provided in the articles. We used the numbers to calculate the pooled sensitivity, specificity, positive likelihood ratio (PLR), negative likelihood ratio (NLR), diagnostic odds ratio (DOR) and corresponding 95% confidence intervals (CIs). The summary receiver operating characteristic (SROC) curve and the area under the SROC curve (AUC) were also calculated to evaluate the pooled diagnostic value of miRNAs. We did not test for the publication bias because only seven articles were ultimately included in this meta-analysis. We used the chi-square and I^2^ tests to access heterogeneity in this study. If *P* < 0.1 or I^2^ > 50%, we defined heterogeneity as significant and would conduct meta-regression, subgroup and sensitivity analyses to discover the sources of heterogeneity. We performed all statistical analyses using Stata 12.0 (StataCorp, College Station, TX, USA). We defined *P*<0.05 as statistically significant.

## Results

### Study characteristics

**Figure 1:**
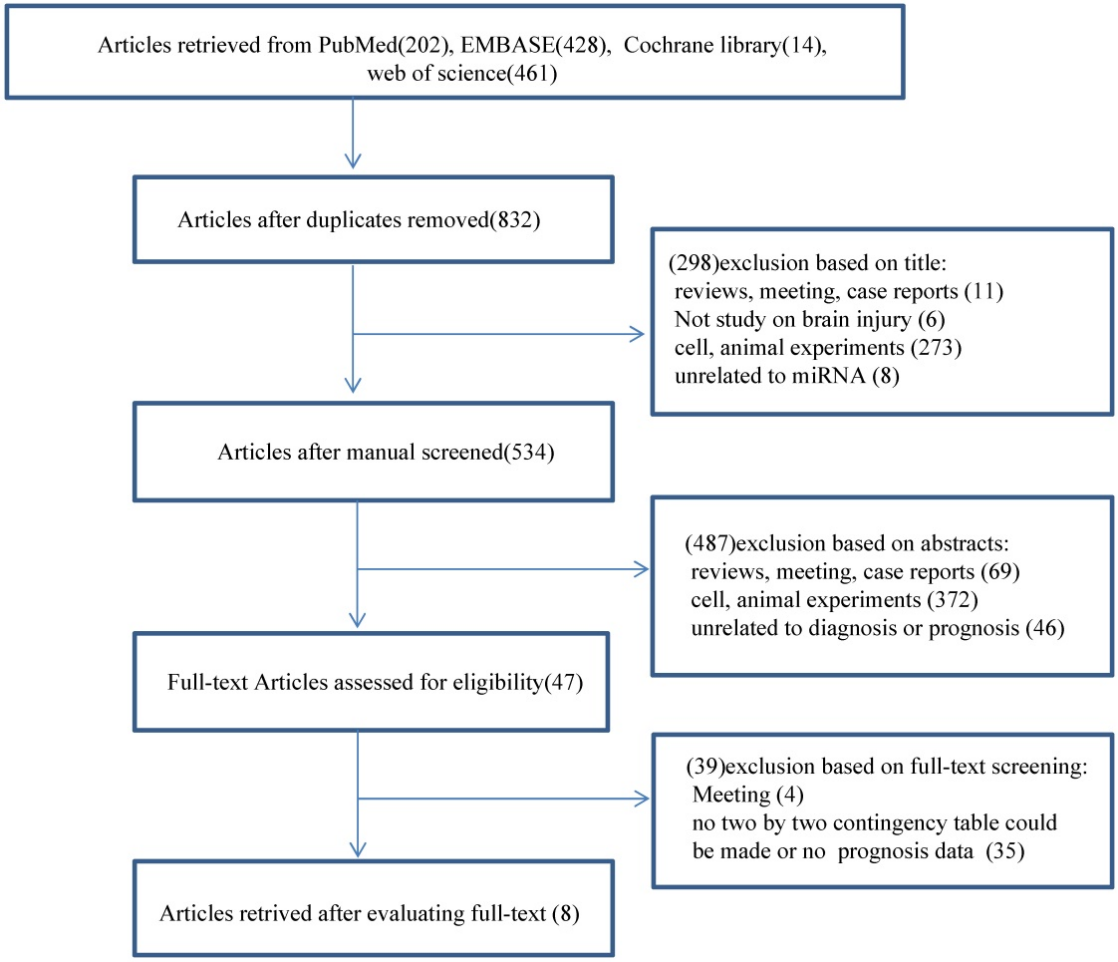
Flow diagram of the study selection for the meta-analysis.

We did a search through PubMed, EMBASE, the Cochrane Library and Web of Science and identified 1105 records. Among these records, 273 were duplicate studies and were, therefore, excluded. We excluded 298 articles after reading the titles and another 487 publications after reviewing the abstracts (**Fig. 1**). The remaining 47 full-text articles were assessed for relevance according to our pre-determined inclusion and exclusion criteria. Subsequently, we excluded 39 publications, including four meetings and 35 without clinical data, and made a two-by-two contingency table to calculate sensitivity, specificity and a corresponding CI. We attempted to calculate pooled hazard ratios (HRs) to analyze the prognostic performance of miRNAs; however, we only extracted enough data to calculate the diagnostic value of miRNAs. Eight diagnostic-related articles were ultimately included in this study 4, 5, 8-13. A flow chart of the selection process for this study presented in **Fig. 1.**

These eight articles included (ranging from the year 2010 to 2020) reported 44 experiments, including different single miRNAs and panel miRNAs (**Tables 1 & 2**). Those articles totally included 215 TBI patients and 152 controls composed of healthy controls and other diseases (**Table 1**). Among the 44 experiments, 29 reported a single miRNA, while the additional 15 discussed a panel miRNAs (**Table 2**). Out of the 44 experiments, ten detected miRNA in plasma, twelve detected miRNA in serum, one detected miRNA in blood and urine, six detected miRNA in saliva, one identified miRNA in brain‐derived extracellular vesicles, and six evaluated the brain tissue. Of the 8 articles, the populations of 6 studies were Caucasian, whereas two studies were Asian. 24 experiments conducted in severe TBI patients, eleven in mild TBI patients, and the remaining nine experiments focused on TBI patients.

### Diagnosis

**Figure 2:**
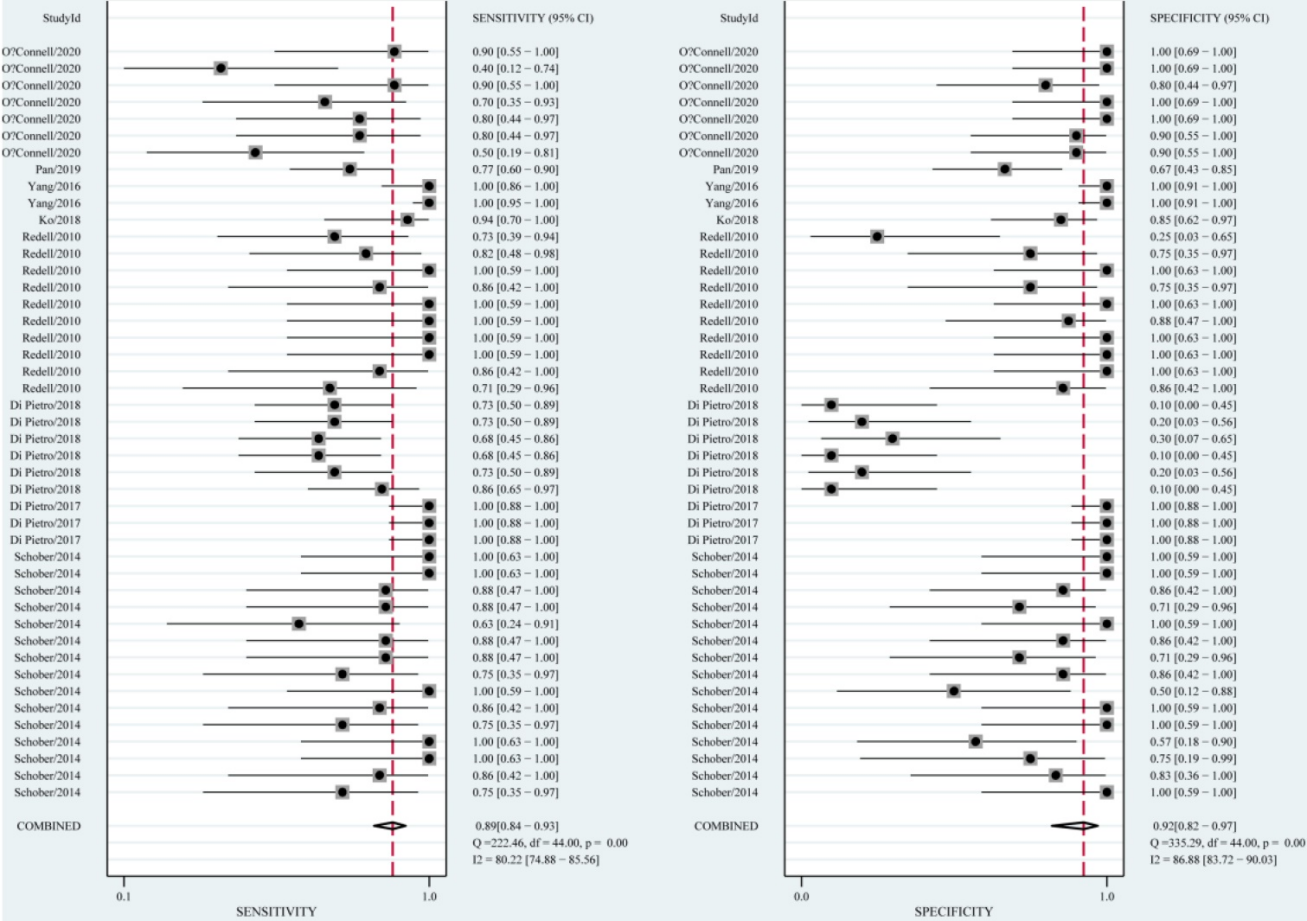
Forest plots for studies on overall miRNAs used in the diagnosis of traumatic brain injury (TBI) among 44 experiments included in the meta-analysis.

**Figure 3:**
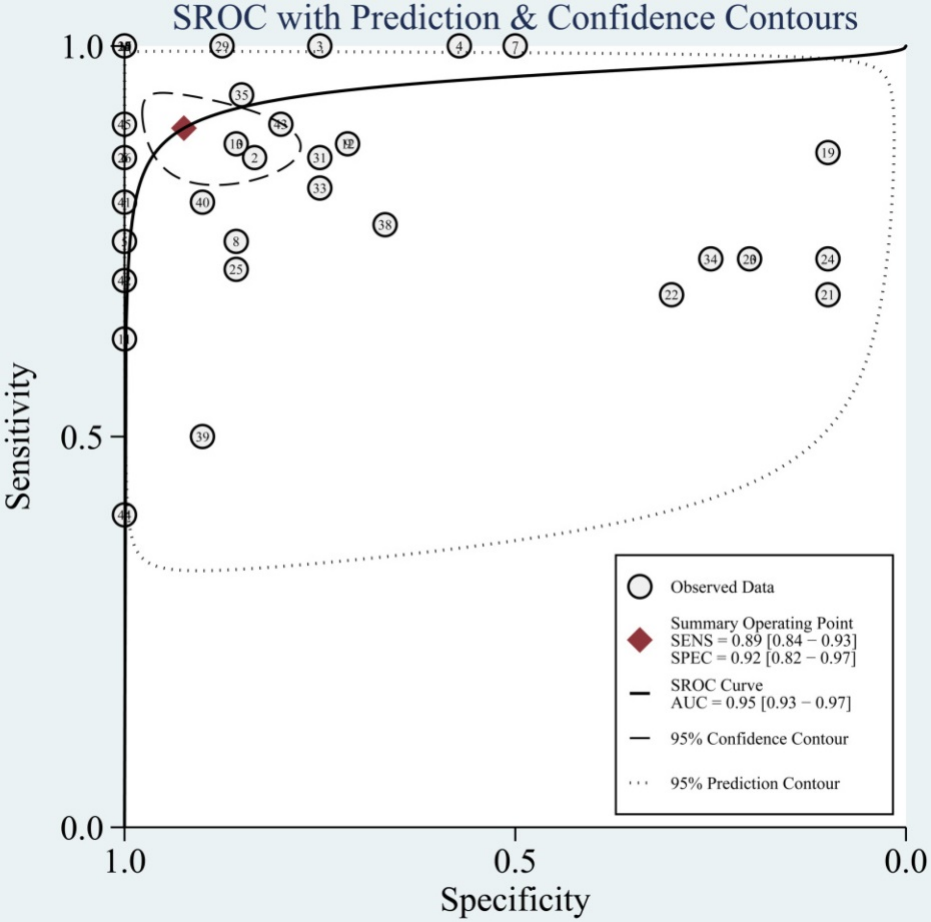
The summary receiver operating characteristic (SROC) curves based on overall miRNAs in the meta-analysis.

**Figure 4:**
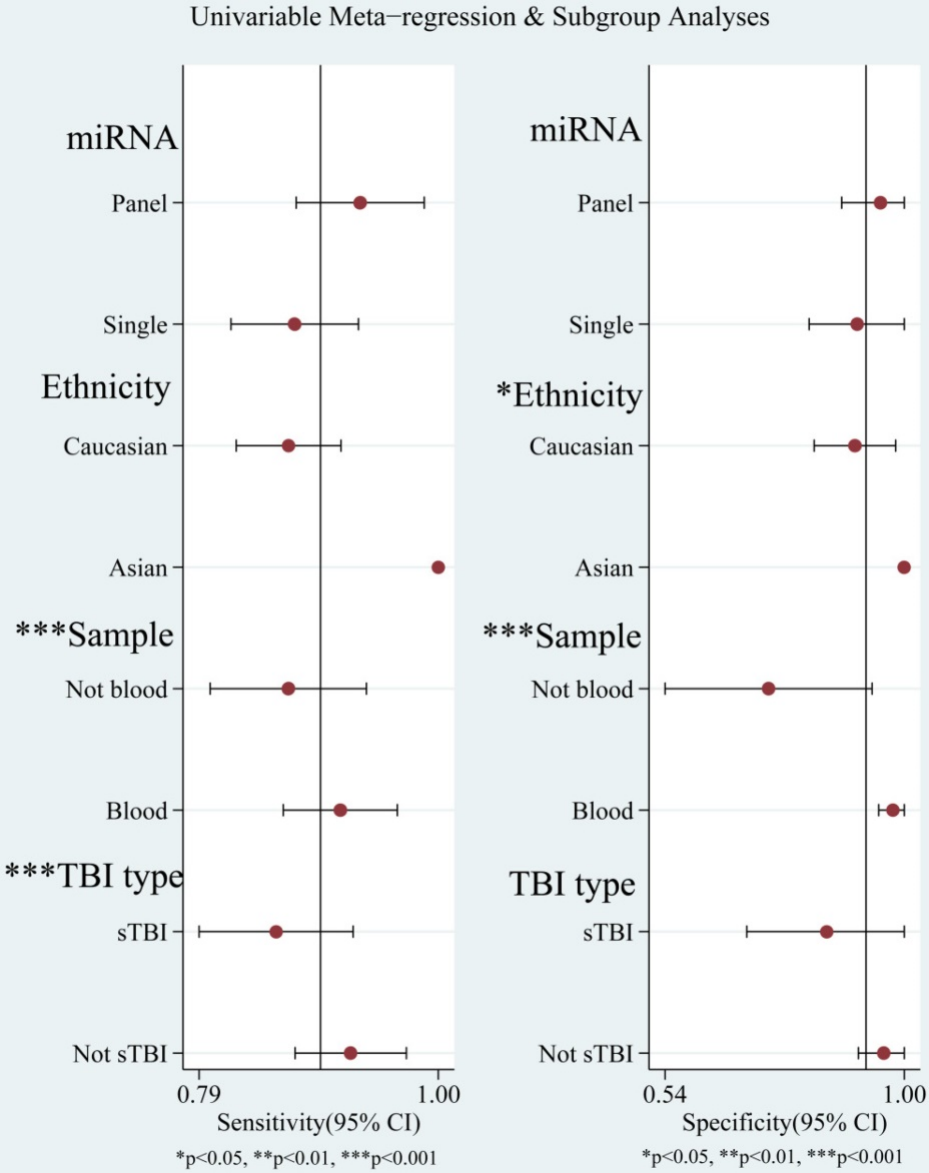
Univariable meta-regression and subgroup analysis for sensitivity and specificity of miRNAs for diagnosis of traumatic brain injury (TBI).

The diagnostic value of miRNAs for all TBI is shown in **Fig. 2**. Forest plots revealed a significant heterogeneity and we, therefore, used the mixed effect model in this meta-analysis. We also summarized sensitivity, specificity, and diagnostic accuracy of all miRNAs in TBI (**Table 3**). The sensitivity, specificity, PLR, NLR, and DOR of overall miRNA for diagnosis of TBI were 0.89 (95% CI: 0.84-0.93), 0.92 (95% CI: 0.82-0.97), 11.8 (95% CI: 4.7-29.6), 0.11 (95% CI: 0.07-0.18) and 103 (95% CI: 30-355). Diagnostic accuracy was evaluated by plotting the summary receiver operating characteristic (SROC) curve (**Fig. 3**). The diagnostic accuracy of overall miRNAs was outstanding since the area under the Curve (AUC) was 0.95 (95% CI: 0.93-0.97). We performed subgroup analyses according to ethnicity, detected sample, miRNA profiling and type of TBI in order to find the heterogeneity (**Fig. 4**). The diagnostic value of single miRNAs was as follows: sensitivity, 0.88; specificity, 0.91; PLR, 9.7; NLR, 0.13; DOR, 74; and AUC, 0.94 (**Fig. S1, Table 3**). However, miRNA panels have a higher overall diagnostic accuracy: sensitivity, 0.93; specificity, 0.95; PLR, 18.3; NLR, 0.08; DOR, 239; and AUC, 0.97 (**Fig. S2, Table 3**). The sensitivity, specificity, PLR, NLR, DOR and AUC of saliva, brain tissue, and blood were 0.73, 0.17, 0.90, 1.59, 1.0 and 0.40; 0.88, 0.87, 7.0, 0.13, 52 and 0.94; 0.94, 0.98, 46.0, 0.06, 756 and 0.99, respectively (**Fig. S3-S5 & Table 3**). This result suggested that miRNAs detected in blood have the highest overall diagnostic accuracy. In the severe TBI patients, the results were 0.91 for sensitivity, 0.92 for specificity, 11.9 for PLR, 0.09 for NLR, 129 for DOR, and 0.97 for AUC (**Fig. S6, Table 3**). The diagnostic value of miRNAs in Caucasians was as follows: sensitivity, 0.88; specificity, 0.91; PLR, 9.7; NLR, 0.14; DOR, 70; and AUC, 0.93 (**Fig. S7, Table 3**).

### Sensitivity analysis, meta-regression analysis and subgroup analysis

**Figure 5:**
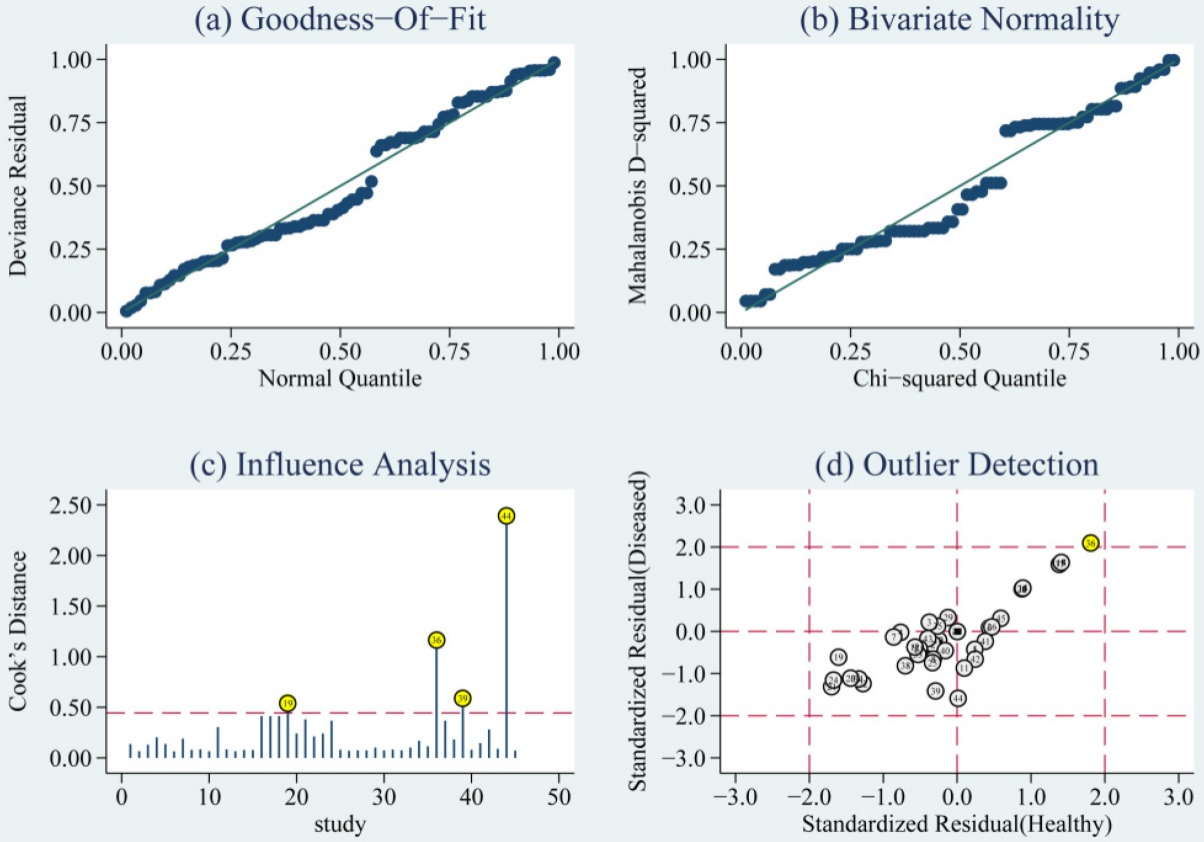
Diagram of (a) Goodness-of-fit, (b) Bivariate normality, (c) Influence analysis, and (d) Outlier detection. Goodness-of-fit and Bivariate normality showed that random effects bivariate model is suitable. Influence analysis identified that studies of Pietro et al, Yang et al. and O'Connell et al were the most dominant studies in weight. Outlier detection implied that no research is the reason for heterogeneity.

The goodness of fit and bivariate normality analyses revealed that the random effects bivariate model was best suited for sensitivity analysis (**Fig. 5a & 5b**). Influence analysis showed that studies of Di Pietro et al., Yang et al., and O'Connell et al. were the leading researches in weight (**Fig. 5c**). Outlier detection identified that no research would significantly affect the heterogeneity of our meta-analysis (**Fig. 5d**). Considering the bias of miRNAs, ethnicity, type of TBI and the detected sample, we conducted a meta-regression analysis and found that the detected sample may influence sensitivity and specificity. Results on subgroup analyses indicated that miRNA detected in blood exhibit the highest heterogeneity compared to brain tissue and saliva. We further conducted a subgroup analysis according to different types of TBI. In sTBI, no apparent heterogeneity was found because the I^2^ value was only 27.07% for sensitivity and 47.04% for specificity (**Fig. S8**). However, we did not do a subgroup analysis of mTBI due to the limitation of the number of mTBI studies.

## Discussion

As potential biomarkers, miRNAs have been clinically tested for the diagnosis of diverse human diseases. In recent years, more researches have determined the diagnostic value of circulating miRNAs for TBI. However, the diagnostic performance of miRNAs in these studies remains controversial. For example, miR-135b acts as a biomarker in the diagnosis of sTBI, with specificity and sensitivity levels of 75% and 86% 11. However, the sensitivity and specificity of mir-135b-5p from saliva samples were 73% and 20% 8. Therefore, the reliability of miRNAs for the diagnosis of TBI remains to be confirmed, particularly with regards to sample type. We conducted this study to systematically assess the accuracy of circulating miRNAs in the diagnosis of TBI.

This meta-analysis involved seven articles, including 215 TBI patients and 152 controls. Our results implied that miRNAs had high sensitivity (0.89) and specificity (0.92) in TBI diagnosis. The pooled PLR was 11.8, suggesting that positive miRNA testing improved the diagnostic probability of TBI by 11.8-fold. Besides, the NLR was 0.11, indicating that negative miRNA testing increased the likelihood of TBI by 89%. A DOR of 1 indicates that miRNAs could not distinguish TBI from control, the DOR of 103 in our article indicated that miRNAs are distinguished biomarkers in the diagnosis of TBI.

**Figure 6:**
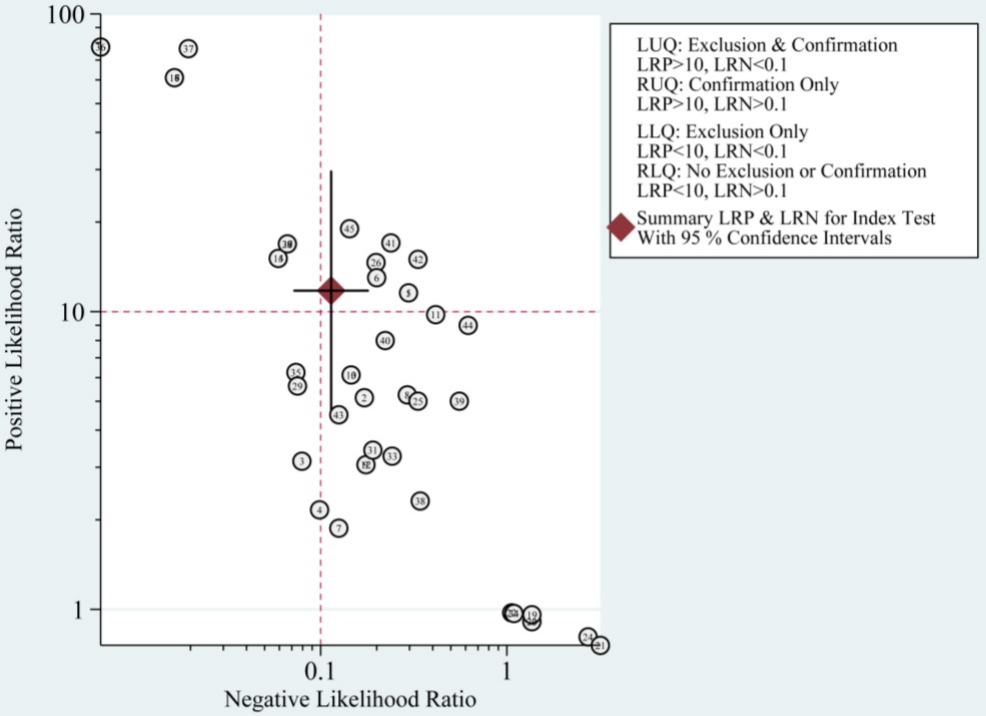
Summary of positive likelihood ratio and negative likelihood ratio for diagnosis of traumatic brain injury (TBI).

**Figure 7:**
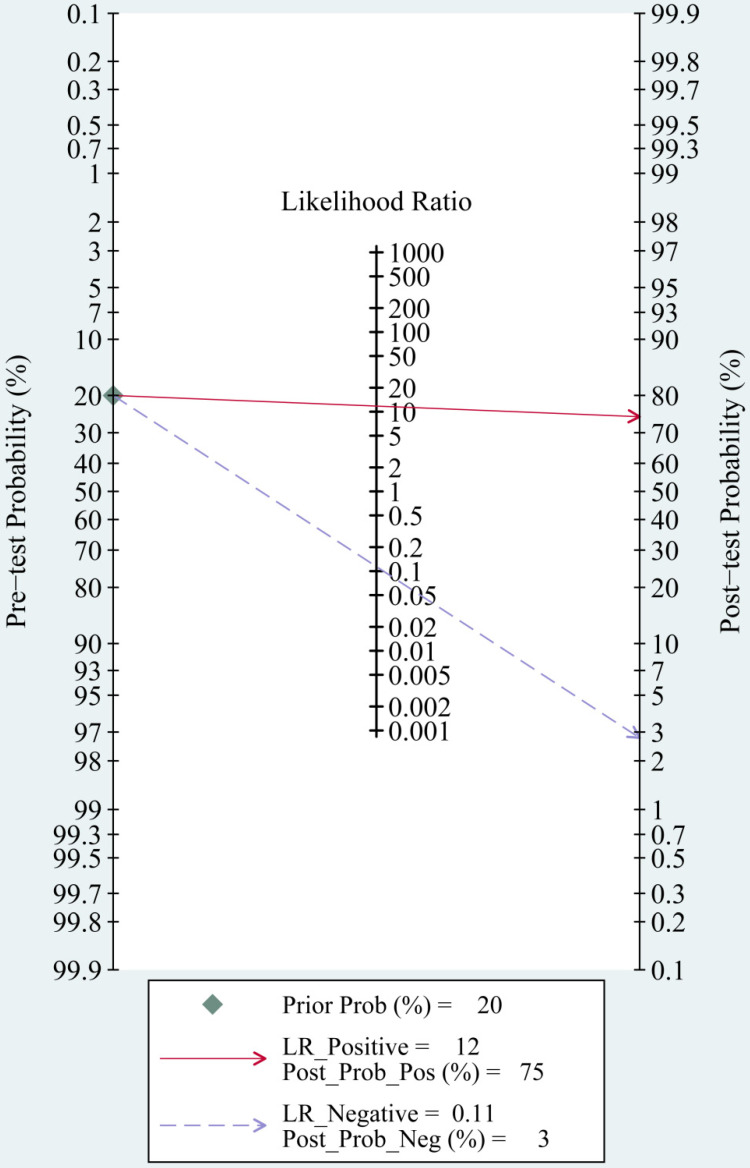
Fagan nomogram of the miRNAs test for diagnosis of traumatic brain injury (TBI).

The most significant role of biomarkers is to help clinicians in clinical decision making. Through likelihood ratios and post-test probabilities, doctors can know the likelihood that a patient has TBI or not. Positive likelihood ratios and negative likelihood ratios were also summarized to assess diagnostic applicability of miRNAs (**Fig. 6**). NLR < 0.1 and PLR > 10 imply a high diagnostic accuracy 7. The articles of Schober et al., Di Pietro et al., and Yang et al. revealed that some miRNAs had outstanding diagnostic accuracy, including single miRNA (miRNA-93, miRNA-425-5p, and miRNA-502) and a panel of miRNAs (miR-138 and miR-744; miR-195 and miR-324-5p). When we set the pretest probability at 20%, a positive likelihood ratio improves the post-test probability to 75%. When the clinician's accuracy rate for diagnosis of TBI based on the patient's symptoms and signs are 20%, the accuracy rate can be increased to 75% by combining miRNAs. When negative likelihood ratio was set at 0.11, the post-test probability for a negative test result is 3%, which means miRNAs can help clinicians reduce the false negative rate (**Fig. 7**).

Notably, ideal biomarkers should be readily measurable in easily accessible samples such as blood or saliva. In our study, miRNAs in blood showed higher diagnostic accuracy, with a sensitivity of 0.94, a specificity of 0.98 and AUC of 0.99. We hypothesise the reason may be that brain specific miRNAs in exosomes can diffuse into the blood once TBI-induced disruption of blood brain barrier has occurred, which can maintain stability and replicability of miRNA from human blood 14. However, miRNA detected in blood, including ten in plasma, twelve in serum, one in blood, showed the highest heterogeneity, we then did a subgroup analyses of plasma and found the I^2^ value in sensitivity and specificity only decreased 21.88% and 7.02% compared to other blood sample (**Fig. S9 and Fig. S10**). Thus, the heterogeneity could not be completely explained by this subgroup analyses. Compared to single miRNA, we demonstrated that multiple-miRNAs have higher diagnostic accuracy for TBI, which was consistent with the findings in other disorders 7, 15-17. We also did a subgroup analysis of sTBI because we combined studies with different TBI severity. After excluding non-severe TBI studies, the I^2^ value in sensitivity and specificity dramatically decreased 53.15% and 39.84%. We thought that non-severe TBI studies could be part of the source of heterogeneity. Pooled sensitivity, pooled specificity and ROC revealed miRNAs have potential diagnostic value for sTBI. We failed to do a subgroup analysis of mTBI because only four of our included experiments reported on the diagnostic value of miRNAs in mTBI patients. We also intend to evaluate the clinical value of miRNAs expression in mTBI patients. Our results were, however, not conclusive because of the limited number of studies. We suggest that more future researchers could explore the clinical value of miRNAs in mTBI.

This meta-analysis has several limitations. Firstly, the results of subgroup analysis, such as blood sample and non-severe TBI, could only find a part of the source of heterogeneity in our study. Second, our meta-analysis had a small sample size. Third, the overall diagnostic accuracy may be exaggerated because studies with positive results have high possibility of publication. Finally, we included studies only written in English, which may bring some bias to our findings.

## Conclusion

Our meta-analysis is the first to evaluate the clinical value of miRNAs expression in TBI patients. miRNAs have potential diagnostic value for TBI. Subgroup analysis demonstrated that miRNAs in blood could improve diagnostic accuracy. Compared to a single miRNA, panels of multiple miRNAs could more accurately identify TBI patients. However, we need include large-sizes researches in future to validate our results and confirm the clinical value of miRNAs in the diagnosis of TBI.

## Supplementary Material

Supplementary figures.

## Figures and Tables

**Table 1:** Characteristics of studies included in meta-analysis

First author	Publish year	Ethnicity	Patients	Controls	Patients/controls	miRNAS	Detected sample
Schober	2014	Caucasian	Severe TBI	Non-TBI	8/7	miR-138, miR-504, miR-16, miR-376a, miR-195, miR-370, miR-320b, miR-135b; miR-744, miR-324-5p, miR-455-3p, let-7a, miR-193b.	Cerebellar tissue
Di Pietro	2017	Caucasian	Mild TBI	healthy volunteers	30/30	miR-425-5p, miR-502, miR-335	Serum
Di Pietro	2018	Caucasian	Concussion	healthy volunteers	22/10	let-7i-5p, miR-142-3p, miR-107, miR-27b-3p, miR-135b-5p.	Salivary
Redell	2010	Caucasian	Severe TBI/mild TBI	healthy volunteers/orthopedic injury patients.	18/16	miR-16, miR-92a, miR-765.	Plasma
Ko	2018	Caucasian	TBI	Healthy controls	16/20	miR‐203b‐5p, miR‐203a‐3p, miR‐206, miR‐185‐5p	EV
Yang	2016	Asian	TBI	Healthy controls	76/38	miR-93	serum
Pan	2019	Asian	TBI	Non-TBI	35/21	miR-155	Blood and urine
O'Connell	2020	Caucasian	TBI	Healthy controls	10/10	miR-124-3p, miR-219a-5p, miR-9-5p, miR-9-3p, miR-137, and miR-128-3p	Serum

TBI: traumatic brain injury; EV: brain‐derived extracellular vesicles.

**Table 2:** False and true positives and negatives of total 44 experiments from 8 included articles

First author	Year	miRNA(s)	TP	FP	FN	TN
**Single miRNA**						
Schober	2014	miR-138	6	0	2	7
Schober	2014	miR-504	6	1	1	5
Schober	2014	miR-16	8	1	0	3
Schober	2014	miR-376a	8	3	0	4
Schober	2014	miR-195	6	0	2	7
Schober	2014	miR-370	6	0	1	7
Schober	2014	miR-320b	7	3	0	3
Schober	2014	miR-135b	6	1	2	6
Schober	2014	miR-744	7	2	1	5
Schober	2014	miR-324-5p	7	1	1	6
Schober	2014	miR-455-3p	5	0	3	7
Schober	2014	let-7a	7	2	1	5
Schober	2014	miR-193b	7	1	1	6
Di Pietro	2017	miR-425-5p	30	0	0	30
Di Pietro	2017	miR-502	30	0	0	30
Di Pietro	2017	miR-335	30	0	0	30
Di Pietro	2018	let-7i-5p	19	9	3	1
Di Pietro	2018	miR-142-3p	16	8	6	2
Di Pietro	2018	miR-107	15	9	7	1
Di Pietro	2018	miR-27b-3p	15	7	7	3
Di Pietro	2018	miR-135b-5p	16	8	6	2
Yang	2016	miR-93	76	0	0	38
Yang	2016	miR-93	25	0	0	38
Pan	2019	miR-155	27	7	8	14
O'Connell	2020	miR-124-3p	5	1	5	9
O'Connell	2020	miR-219a-5p	8	1	2	9
O'Connell	2020	miR-9-5p	8	0	2	10
O'Connell	2020	miR-9-3p	7	0	3	10
O'Connell	2020	miR-137	9	2	1	8
O'Connell	2020	miR-128-3p	4	0	6	10
**miRNA panel**						
Schober	2014	miR-138,miR-744	8	0	0	7
Schober	2014	miR-195,miR-324-5p	8	0	0	7
Di Pietro	2018	let-7i-5p,miR-142-3p,miR-107,miR-27b-3p,miR-135b-5p.	16	9	6	1
Redell	2010	miR-16,miR-92a	5	1	2	6
Redell	2010	miR-16,miR-765	6	0	1	8
Redell	2010	miR-92a,miR-765	7	0	0	8
Redell	2010	miR-92a,miR-765,miR-16,	7	0	0	8
Redell	2010	miR-16,miR-92a	7	1	0	7
Redell	2010	miR-16,miR-765	7	0	0	8
Redell	2010	miR-92a,miR-765	6	2	1	6
Redell	2010	miR-92a,miR-765,miR-16	7	0	0	8
Redell	2010	miR-16,miR-92a	9	2	2	6
Redell	2010	miR-16,miR-92a	8	6	3	2
Ko	2018	miR-203b-5p,miR-203a-3p,miR-206,miR-185-5p.	15	3	1	17
O'Connell	2020	miR-124-3p, miR-219a-5p, miR-9-5p, miR-9-3p, miR-137, and miR-128-3p.	9	0	1	10

TP: true positive; FP: false positive; FN: false negative; TN: true negative.

**Table 3:** Summary of diagnostic value of miRNAs for diagnosis of TBI

miRNAs	sensitivity	specificity	PLR	NLR	DOR	AUC
overall	0.89 (0.84-0.93)	0.92 (0.82-0.97)	11.8 (4.7-29.6)	0.11 (0.07-0.18)	103 (30-355)	0.95 (0.93-0.97)
Single miRNA	0.88 (0.81-0.93)	0.91 (0.76-0.97)	9.7 (3.4-27.9)	0.13 (0.08-0.23)	74 (18-308)	0.94 (0.92-0.96)
miRNA panels	0.93 (0.84-0.97)	0.95 (0.74-0.99)	18.3(3.0-111.9)	0.08 (0.03-0.19)	239(21-2691)	0.97 (0.95-0.98)
Blood	0.94 (0.85-0.98)	0.98 (0.90-1.00)	46.0 (8.9-238.1)	0.06 (0.02-0.16)	756 (79-7255)	0.99 (0.98-1.00)
Brain tissue	0.88 (0.79-0.94)	0.87 (0.75-0.94)	7.0 (3.5-14.1)	0.13 (0.08-0.24)	52 (20-124)	0.94 (0.91-0.96)
Salivary	0.73 (0.65-0.80)	0.17 (0.09-0.28)	0.9 (0.8-1.0)	1.59 (0.85-3.00)	1.0 (0.0-1.0)	0.40 (0.36-0.45)
Caucasians	0.88 (0.82-0.92)	0.91 (0.80-0.96)	9.7 (4.0-23.2)	0.14 (0.09-0.21)	70 (22-222)	0.93 (0.90-0.95)
sTBI	0.91 (0.85-0.95)	0.92 (0.84-0.96)	11.9 (5.6-25.6)	0.09 (0.05-0.17)	129 (45-374)	0.97 (0.95-0.98)

PLR: positive likelihood ratio; NLR: negative likelihood ratio; DOR: diagnostic odds ratio; AUC: area under the curve; sTBI: severe traumatic brain injury.

## Abbreviations

miRNAs: MicroRNAs
AUC: area under the curve
CI: confidence interval
SROC: summary receiver operator characteristic
TP: true positive
FP: false positive
FN: false negative
TN: true negative
PLR: positive likelihood ratio
NLR: negative likelihood ratio
DOR: diagnostic odds ratio
TBI: traumatic brain injury
mTBI: mild traumatic brain injury
sTBI: severe traumatic brain injury
HRs: hazard ratios
