# Supplementary figures and images for: MicroRNAs as potential biomarkers for the diagnosis of Traumatic Brain Injury: A systematic review and meta-analysis
Source: Int J Med Sci. 2021 Jan 1;18(1):128–36. doi: 10.7150/ijms.48214 (PMC7738974; doi:10.7150/ijms.48214)

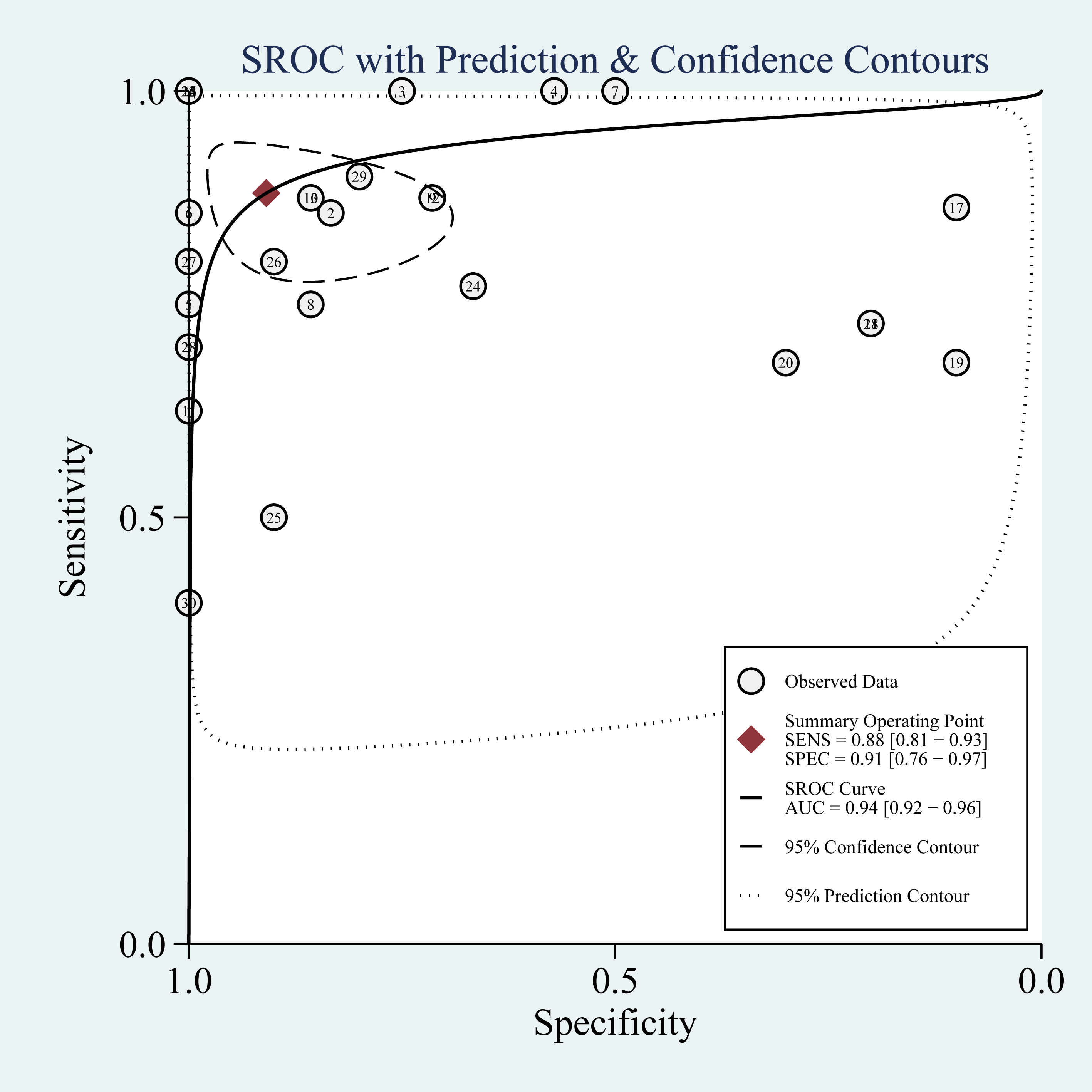

Supplement: Supplementary file 1 — Supplementary figures. [file ijmsv18p0128s1.zip › Supplementary materials/Fig.S1.tif]

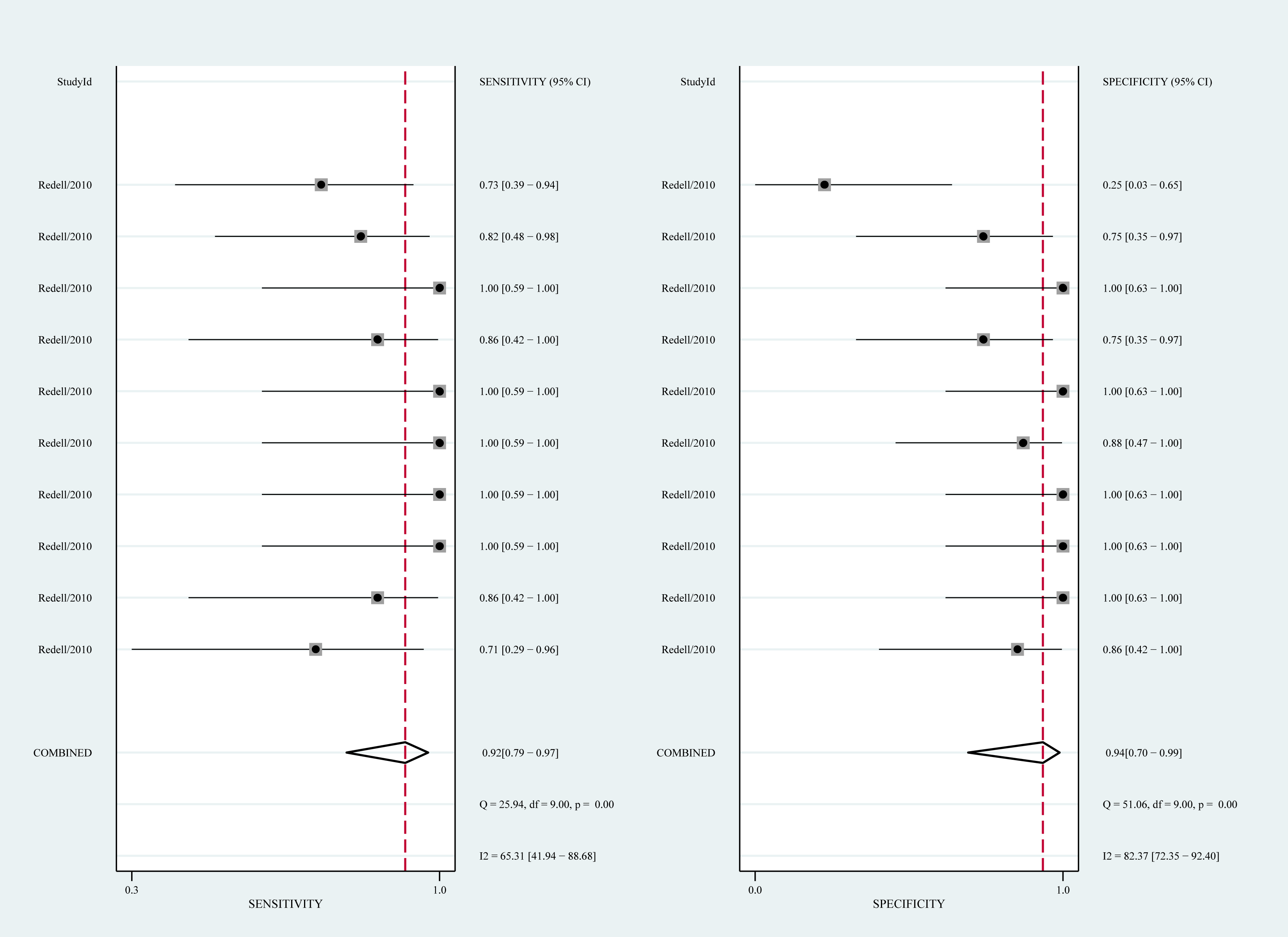

Supplement: Supplementary file 1 — Supplementary figures. [file ijmsv18p0128s1.zip › Supplementary materials/Fig.S10.tif]

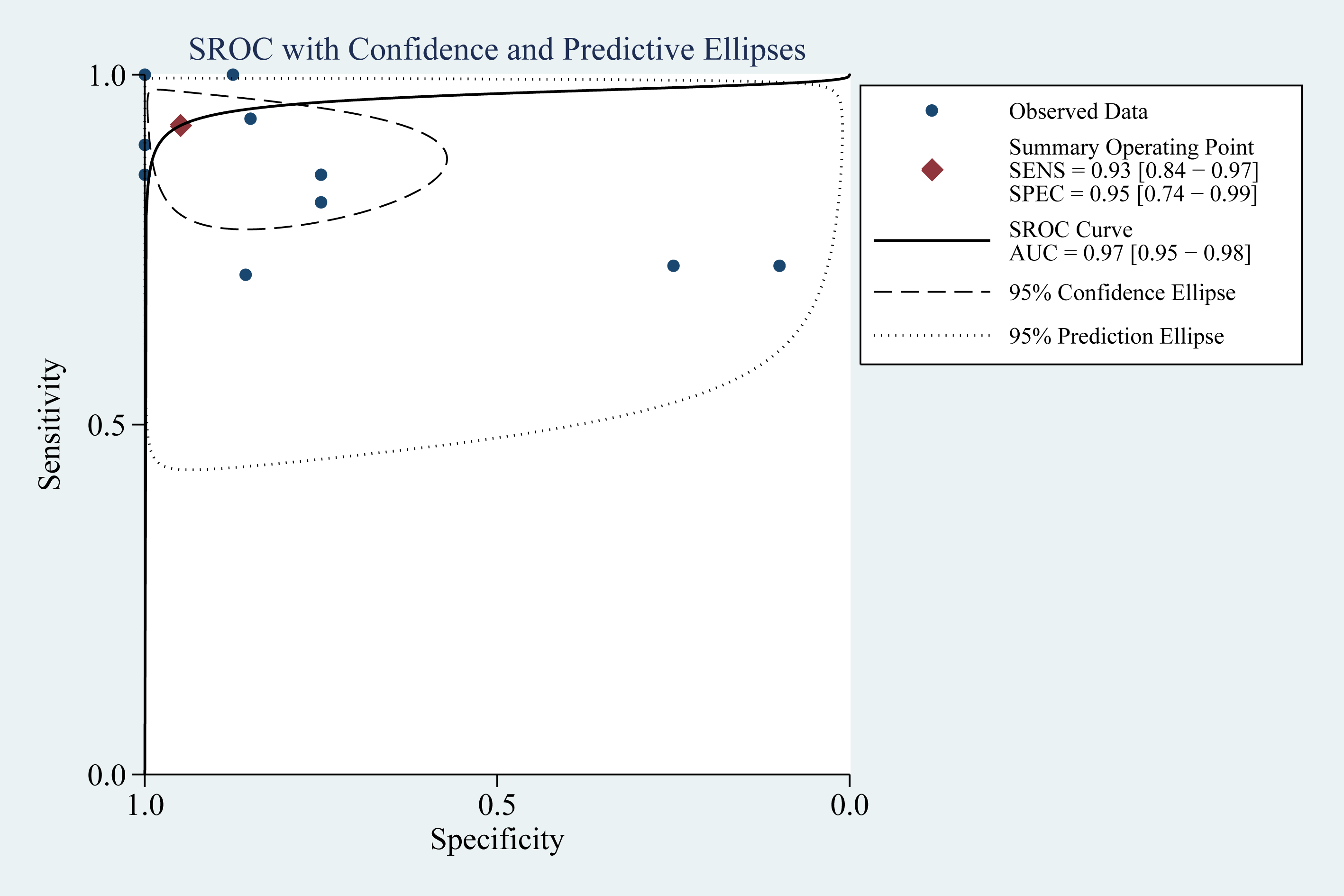

Supplement: Supplementary file 1 — Supplementary figures. [file ijmsv18p0128s1.zip › Supplementary materials/Fig.S2.tif]

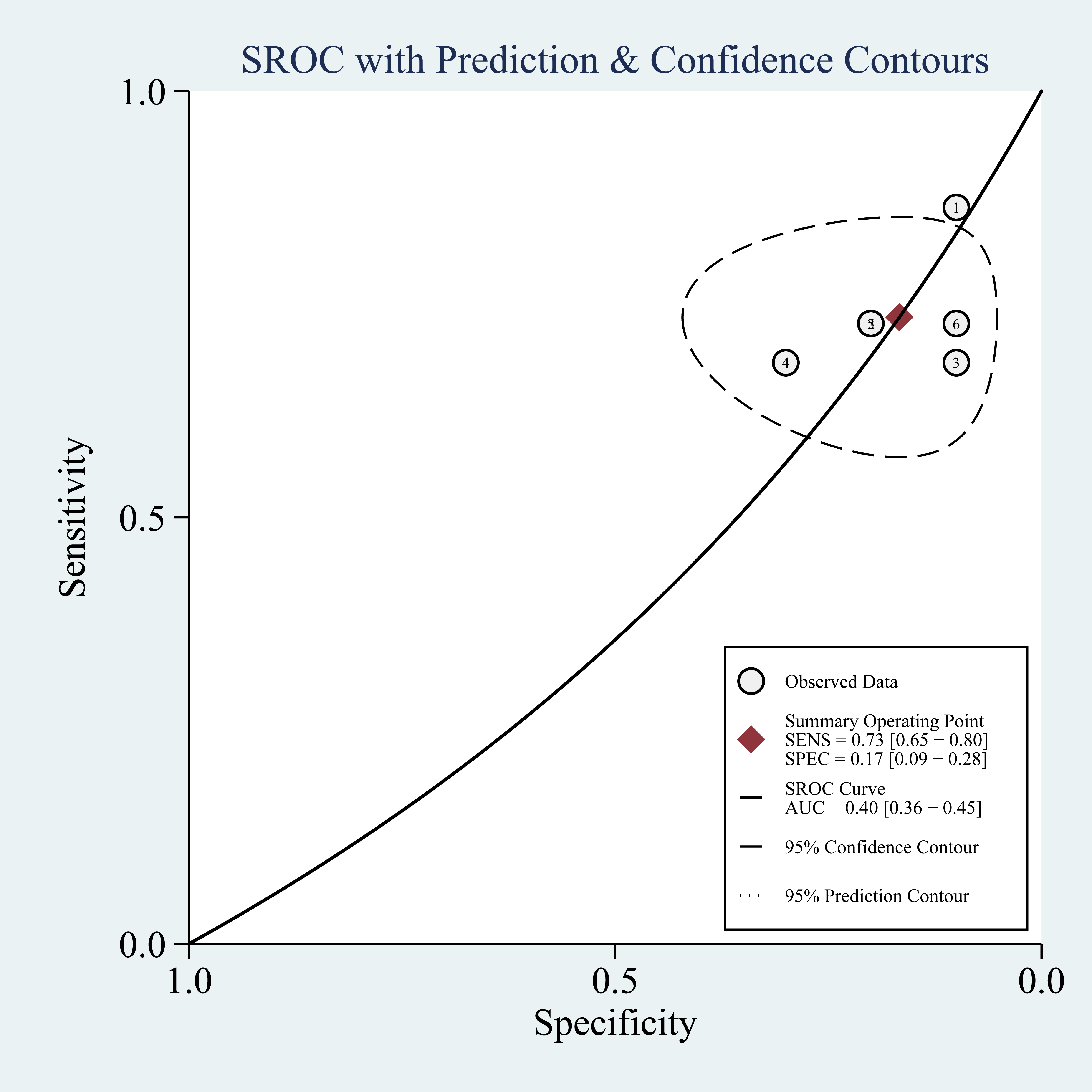

Supplement: Supplementary file 1 — Supplementary figures. [file ijmsv18p0128s1.zip › Supplementary materials/Fig.S3.tif]

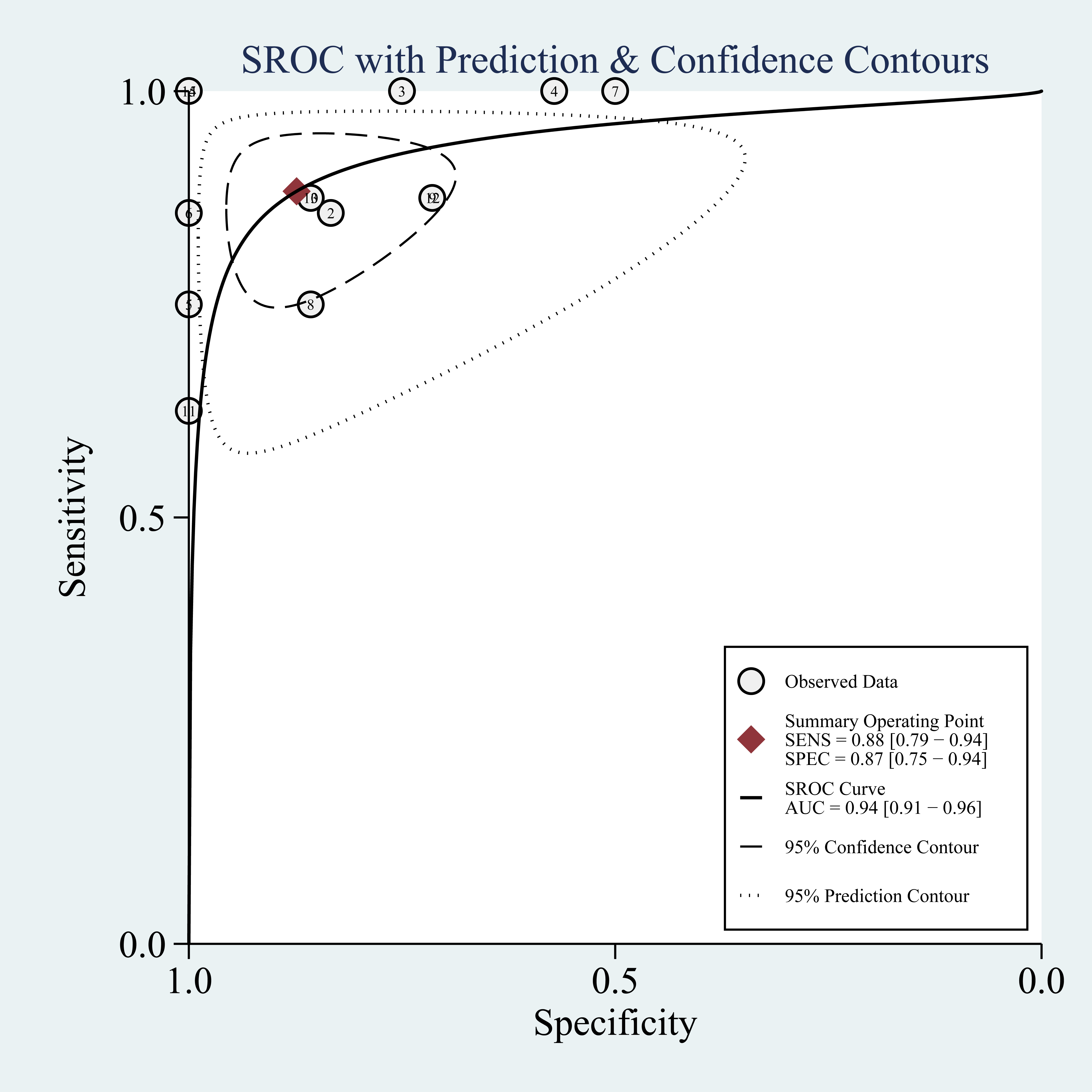

Supplement: Supplementary file 1 — Supplementary figures. [file ijmsv18p0128s1.zip › Supplementary materials/Fig.S4.tif]

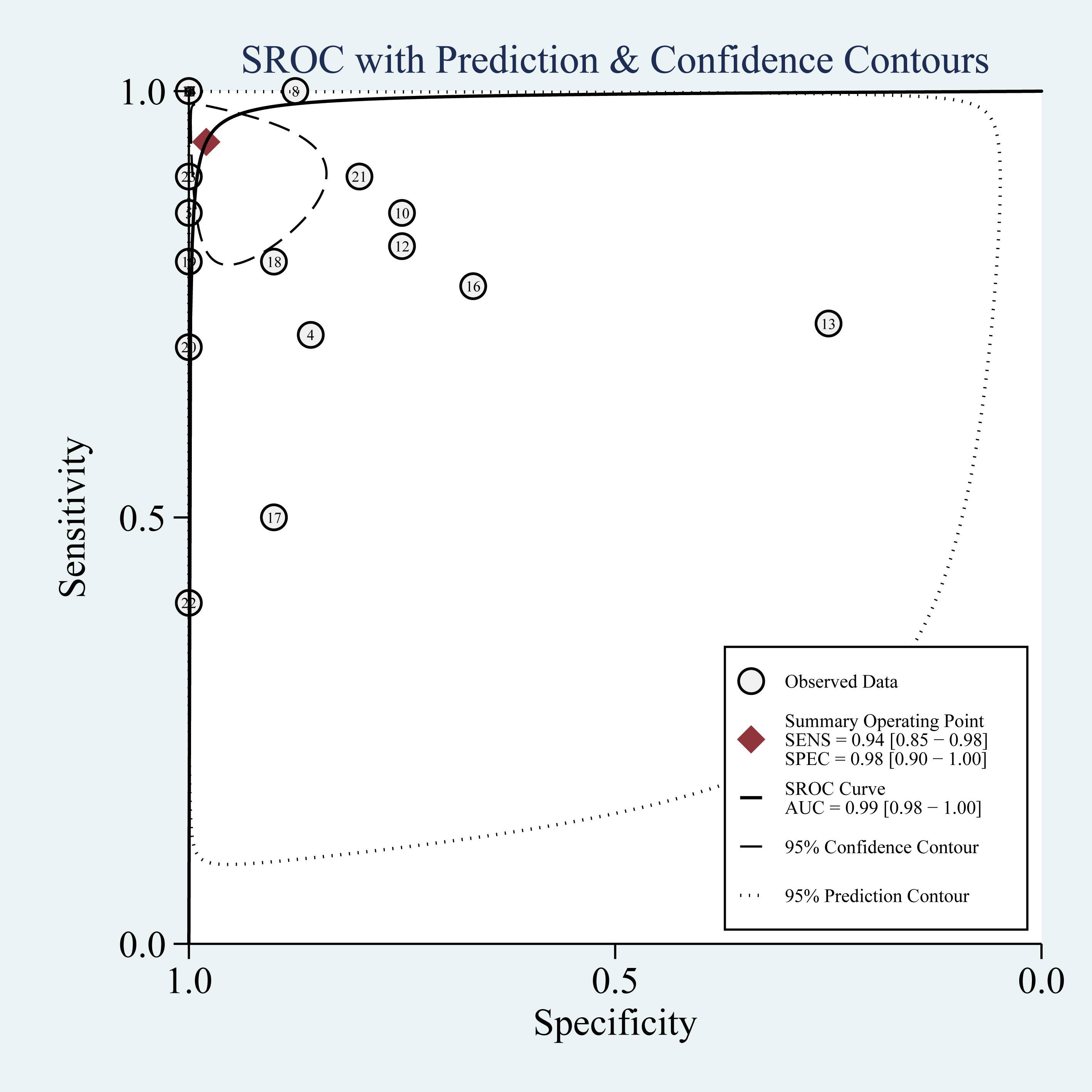

Supplement: Supplementary file 1 — Supplementary figures. [file ijmsv18p0128s1.zip › Supplementary materials/Fig.S5.tif]

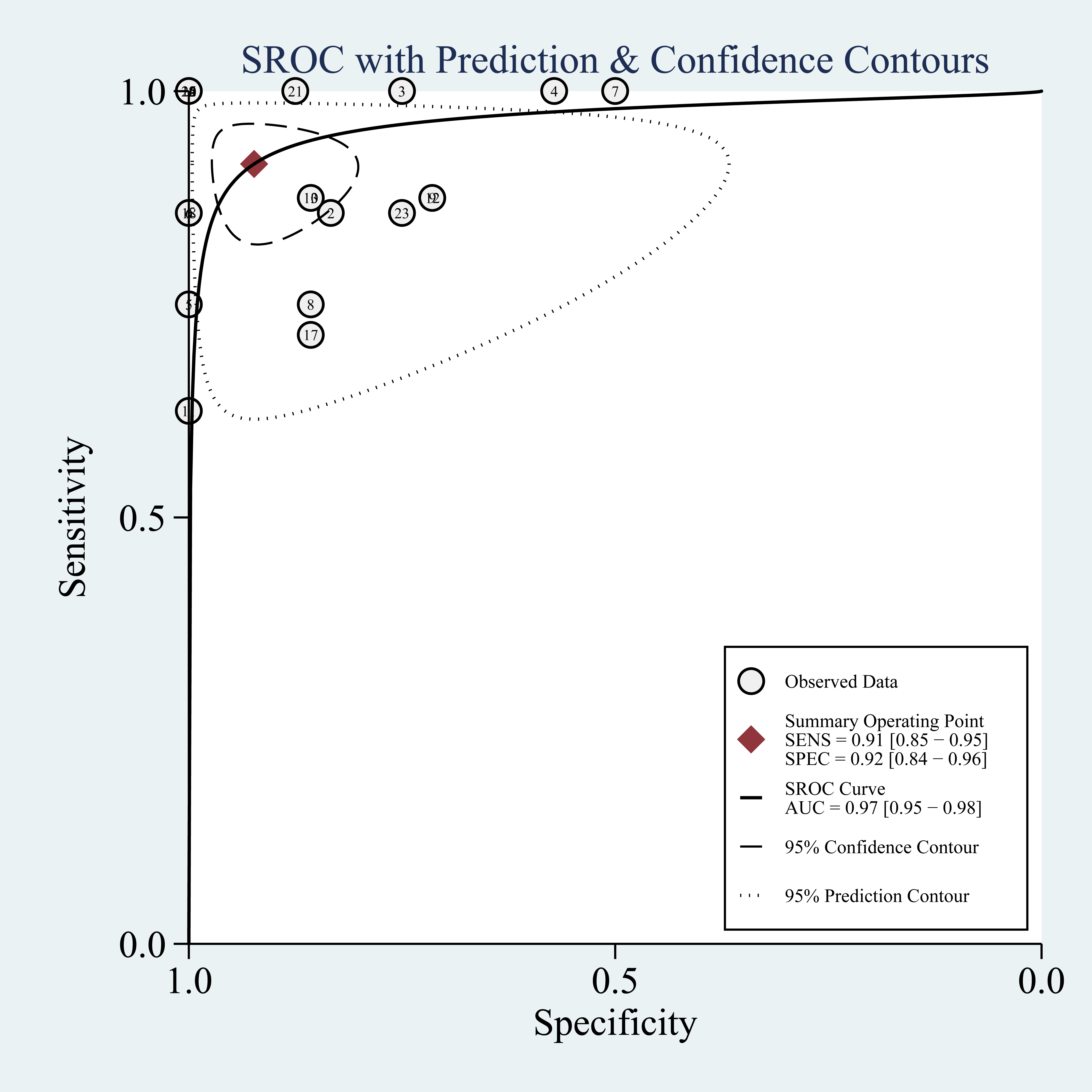

Supplement: Supplementary file 1 — Supplementary figures. [file ijmsv18p0128s1.zip › Supplementary materials/Fig.S6.tif]

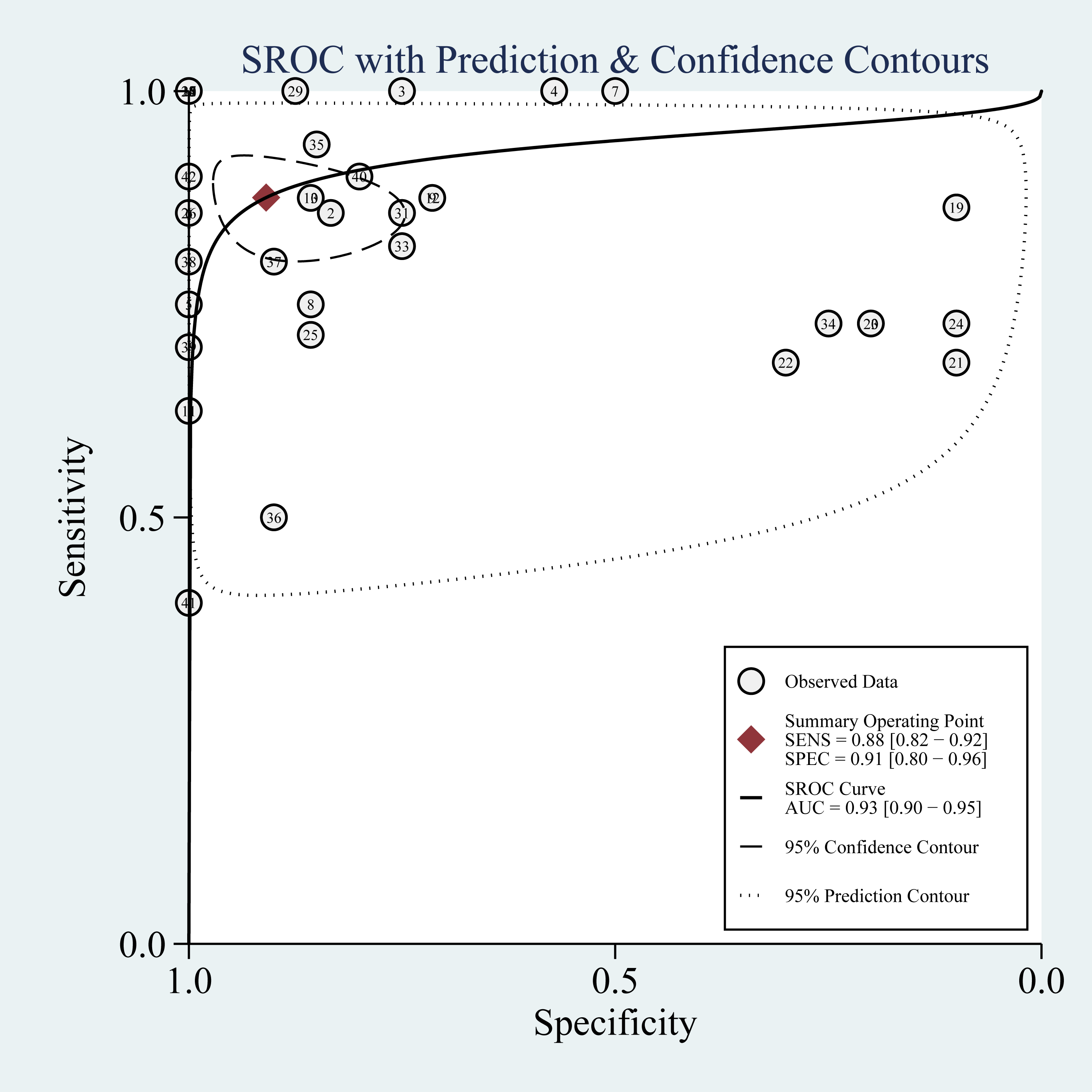

Supplement: Supplementary file 1 — Supplementary figures. [file ijmsv18p0128s1.zip › Supplementary materials/Fig.S7.tif]

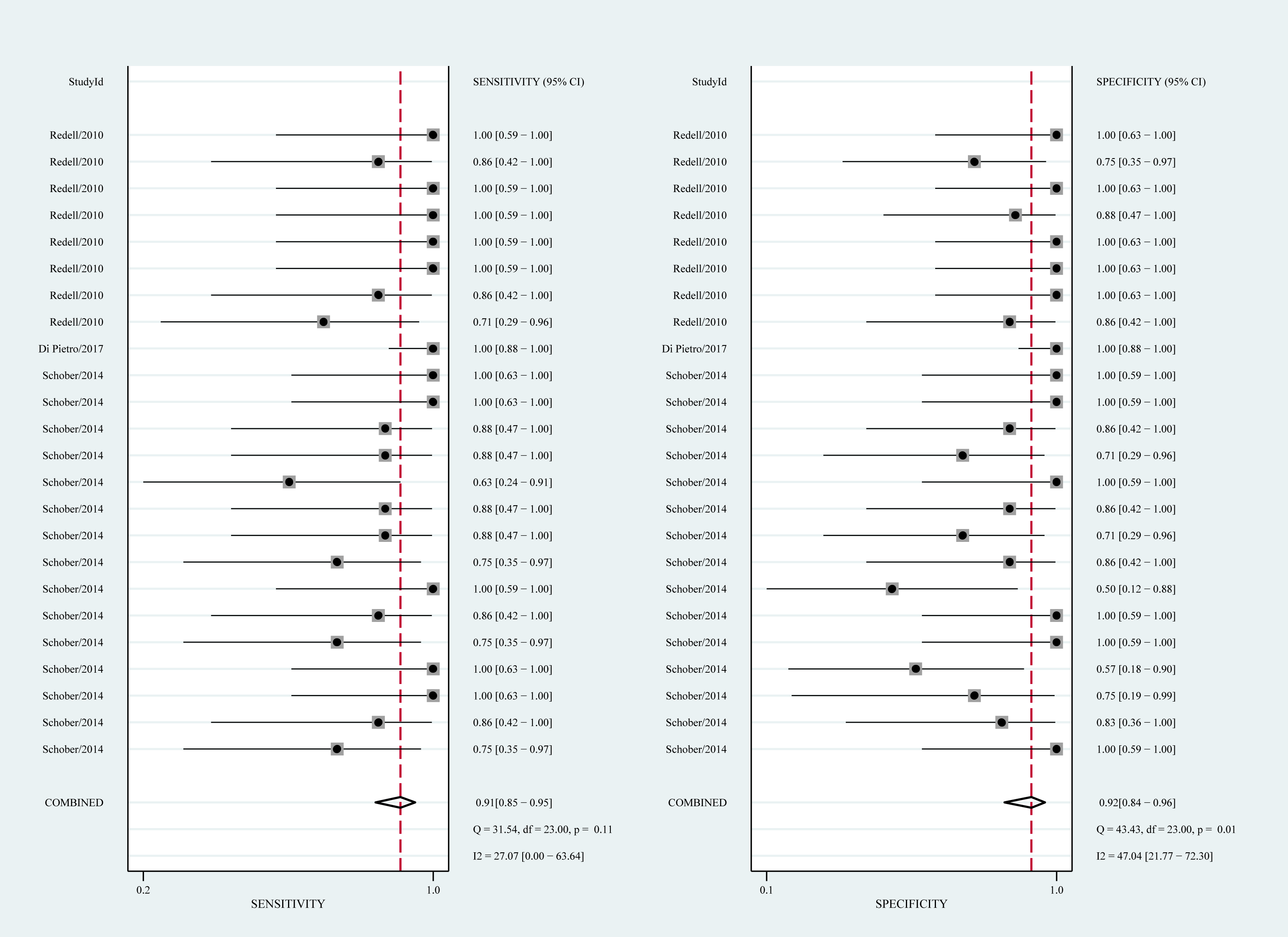

Supplement: Supplementary file 1 — Supplementary figures. [file ijmsv18p0128s1.zip › Supplementary materials/Fig.S8.tif]

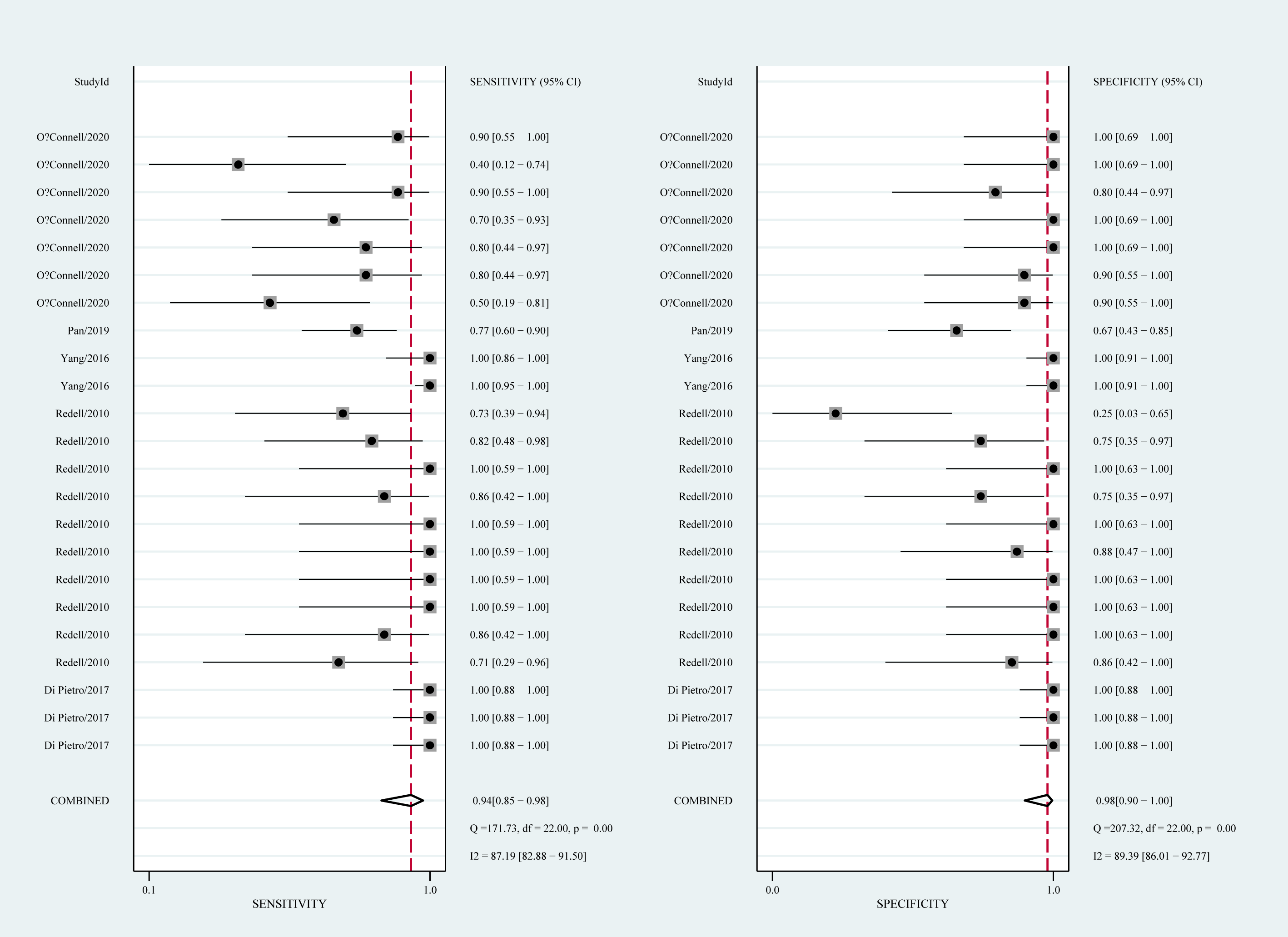

Supplement: Supplementary file 1 — Supplementary figures. [file ijmsv18p0128s1.zip › Supplementary materials/Fig.S9.tif]
